# Functional hypogonadism in adolescence: an overlooked cause of secondary hypogonadism

**DOI:** 10.1530/EC-23-0190

**Published:** 2023-09-27

**Authors:** Rebeca Esquivel-Zuniga, Alan D Rogol

**Affiliations:** 1 Department of Pediatrics, University of Virginia, Charlottesville, Virginia, USA

**Keywords:** functional hypogonadotropic hypogonadism, puberty, delayed puberty, chronic illness, sex steroids, growth

## Abstract

Hypogonadism is a clinical syndrome resulting from failure to produce physiological concentrations of sex steroid hormones with accompanying symptoms, such as slowed growth and delayed pubertal maturation. Hypogonadism may arise from gonadal disease (primary hypogonadism), dysfunction of the hypothalamic–pituitary axis (secondary hypogonadism) or functional hypogonadism. Disrupted puberty (delayed or absent) leading to hypogonadism can have a significant impact on both the physical and psychosocial well-being of adolescents with lasting effects. The diagnosis of hypogonadism in teenagers can be challenging as the most common cause of delayed puberty in both sexes is self-limited, also known as constitutional delay of growth and puberty (CDGP). Although an underlying congenital cause should always be considered in a teenager with hypogonadism, acquired conditions such as obesity, diabetes mellitus, other chronic diseases and medications have all been associated with low sex steroid hormone levels. In this review, we highlight some forms of functional hypogonadism in adolescents and the clinical challenges to differentiate normal variants from pathological states.

## Introduction

The transition between childhood and (emerging) adulthood is generally referred to as the adolescent period. It is a time when the well-known biological process (pubertal maturation) is accompanied by adaptations in the psychosocial, cultural and intellectual spheres. The biological process depends upon the re-awakening of the hypothalamic–pituitary–gonadal (HPG) axis heralded by the re-emergence of pulsatile GnRH secretion directed by other, upstream, hypothalamic factors including neurons that release kisspeptin, neurokinin B and dynorphin (KNDy) that stimulate pituitary release of gonadotropins, luteinizing hormone (LH) and follicle-stimulating hormone (FSH). This hormonal cascade results in gonadal maturation with subsequent production of sex steroids, non-steroidal factors and gametes (1, 2).

Although the HPG axis awakens in late childhood, the external signs of pubertal maturation: early breast development (Tanner stage II) and an increase in testicular volume (≥4 mL) occur later. That process is considered physiologic if it occurs within 2.0–2.5 s.d. from the mean, which translates in the developed world to 8–13 years in females and 9–14 years in males. Often puberty is completed within 2.5–3 years. One may then consider pubertal maturation to be delayed in boys if the onset is after 14 years and in girls if after 13 years or to be prolonged if the process unfolds over more than 4 years (3, 4).

The differential diagnosis of delayed puberty (DP) is broad (Table 1). The most common etiology is constitutional delay of growth and puberty (CDGP), a non-pathological state in which the maturation of the HPG axis is delayed and pubertal maturation begins at an age at the extreme end of normal. CDGP is a diagnosis of exclusion, and alternative causes of DP should first be considered (5, 6). These can be divided into three main categories: (1) hypergonadotropic hypogonadism, characterized by elevated levels of LH and FSH and due to gonadal failure or the inability to synthesize or respond to sex steroids; (2) permanent hypogonadotropic hypogonadism, characterized by low levels of FSH and LH and can be caused by an abnormality in the central nervous system (CNS) or can be associated with other neuroendocrine deficiencies or be isolated (isolated hypogonadotropic hypogonadism, IHH) or lastly idiopathic; and (3) functional hypogonadotropic hypogonadism (FHH), characterized by low levels of FSH and LH, but which represents a *transient* delay in HPG axis maturation due to an associated condition, such as obesity, inflammatory bowel disease or anorexia nervosa and/or medications such as opioids or glucocorticoids (7, 8). CDGP and FHH are found most often in boys, but hypergonadotropic hypogonadism is found more often in girls (9). Genetic evaluation has helped to distinguish IHH from the more common CDGP (10); however, it remains to be determined if some subjects with FHH will have specific genetic markers for the determination of delayed growth and pubertal maturation.

**Table 1:** Differential diagnosis of delayed puberty in addition to CDGP.

	Hypergonadotropic hypogonadism	Hypogonadotropic hypogonadism	Functional hypogonadotropic hypogonadism
Male	Klinefelter syndrome Congenital anorchia/testicular regression	Kallmann syndrome Combined pituitary hormone deficiency CNS: Tumors/infiltrative diseases Chemotherapy/Radiation therapy	Systemic illness e.g. (inflammatory bowel disease, celiac disease, anorexia nervosa, excessive exercise, chronic kidney disease, sickle cell disease and thalassemia, severe obesity, opioids); chronic stress
Female	Turner syndrome Premature ovarian insufficiency	Kallmann syndrome Combined pituitary hormone deficiency CNS: Tumors/infiltrative diseases Chemotherapy/Radiation therapy	Systemic illness e.g. (inflammatory bowel disease, celiac disease, anorexia nervosa, bulimia nervosa, excessive exercise, chronic kidney disease, sickle cell disease and thalassemia, opioids); chronic stress

The appearance and maintenance of secondary sexual characteristics have a crucial impact on the physical and psychosocial well-being, both in adolescents and emerging adults. Hypogonadism can cause short- and long-term consequences, such as to increase the risk of metabolic syndrome (11, 12) and secondary osteoporosis (13). Adolescents affected by pubertal delay often feel distressed by their condition. This may have an important impact on quality of life, even as emerging adults (14, 15).

In this review, we concentrate on various forms of disrupted puberty that present as functional hypogonadism in adolescents and how one might initiate an evaluation. There are several recent reviews that emphasize the more global issues of the differential diagnosis of adolescents with DP with emphasis on some congenital and acquired forms (3, 9, 16, 17).

## Obesity

Childhood-onset obesity is an increasing concern worldwide. Both pubertal maturation and skeletal growth are sensitive to nutritional status and excessive fat mass (18). Previous studies in adult males have indicated a negative relationship between the degree of obesity and the inhibition of the HPG axis, decreasing concentrations of total and free testosterone. Moreover, those males with type 2 diabetes mellitus (T2DM) and obesity or other components of the metabolic syndrome are more likely to have diminished testosterone levels than those with isolated obesity (19). This association has been identified in obese pubertal boys as well, as they may present with DP, associated with insulin resistance. Higher BMI contributes to lower serum testosterone concentration in pubertal males and suggests that the relative hypogonadism in obese males is related to the degree of insulin resistance. Obese boys who have delayed pubertal maturation are also likely to have lower circulating testosterone concentrations (20, 21).

The etiology of this relative hypogonadism in obese pubertal males remains unclear. Although leptin concentrations rise early in puberty, the subsequent trajectories differ between the sexes: in males, the levels decline as puberty unfolds. However, in girls, the levels increase as they add the gynecoid distribution of adipose tissue.

In obese males, the pattern differs as leptin levels increase and may be responsible in part for the suppression of the HPG axis. An alternative explanation suggests that it is perhaps the increased aromatase activity of the additional adipose tissue that dampens the HPG axis, increasing the conversion of testosterone to estradiol and further suppressing pituitary LH secretion. In the female, insulin resistance plays a role in hyperandrogenemia; however, in males testosterone levels are lower (22, 23).

Recent guidelines from European Academy of Andrology (EAA) recommend lifestyle changes, including physical exercise and weight reduction, as the first line of management in overweight and obese men with functional hypogonadism since weight loss may increase testosterone concentrations (24). Weight loss may be a strategy to improve HPG function; especially in younger males when compared to older males for the rise in total testosterone was greater in the former than the latter (25).

## Eating disorders

Women with functional hypothalamic amenorrhea (FHA) often have hypercortisolemia due to activation of the hypothalamic–pituitary–adrenal (HPA) axis. This condition has been described in amenorrheic athletes (mainly runners), but it also can occur in those who do not exercise intensively (26). Reduced pulsatile release of LH is considered etiological as acute energy deprivation does stimulate the HPA axis (27). Reproduction is a large energy process and thus low energy availability can inhibit that.

Other mechanisms that activate the HPA axis can also inhibit the HPG axis; for example, psychosocial stress (28), some endocrine disrupting chemicals, commonly bisphosphonates and polychlorinated biphenyls (29). One possible treatment for women with FHA is behavioral modification that does decrease the hypercortisolemia permitting the resumption of cyclic ovarian function in some. The exact sequence for this ‘repair’ of the HPG axis includes kisspeptin, its receptor, GPR54 and kisspeptin/neurokinin B/dynorphin neurons (KNDy), which integrate these signals and metabolic ones to either stimulate or inhibit GnRH release (30). Most studies show that this constellation of neuroendocrine alterations is more prevalent in adolescents with disordered eating patterns, including extreme dieting, eating disorders including anorexia, bulimia and excessive physical activity (31). Loucks and colleagues showed that the adolescent HPG axis was far more sensitive to stressors, in this case low energy availability, than that for gynecologically older women (32).

Common clinical manifestations of FHA include DP, amenorrhea, infertility and long-term health consequences of hypoestrogenism in women. As an eating disorder continues with time, it may become more difficult to re-gain cyclic ovarian function with bone loss or failure to accrue more (peak) bone mass being a major long-term consequence along with low bone mineral density and stress fractures, despite the anabolic effect of weight-bearing exercise (33). Repeated stress fractures may occur in up to 30% of ballet dancers (34).

As noted above, amenorrhea may persist long after removal of the causative factors. Nevertheless, regular menses may never resume in some women who have regained their pre-morbid body weight but have not resolved their overall stress level, be that physical or psychological. One study suggested that the weight gain required to restore menses was 2.0 kg greater than the weight at which menses stopped (35). At least 6–12 months of weight stabilization may be required for the resumption of menses. This emphasizes the importance of psychological factors and other forms of stress (36).

## Energy deficiency

Energy deficiency may be a cause of (transient) hypogonadotropic hypogonadism, mimicking the initial work done on the female athlete triad (diminished energy availability, oligo/amenorrhea and diminished bone strength) (37). More recently these concepts have been applied to males as well, leading to the construct of relative energy deficiency in sport (RED-S) (38, 39), a medical condition with deleterious consequences for the metabolic and endocrine systems. At the same time, our adult endocrine colleagues have described young men with a median age of about 20 years, who were mostly underweight with low circulating levels of testosterone and LH, meeting the criteria for (mostly, marked) hypogonadotropic hypogonadism. The defining characteristic of this group was a reversal of the hypogonadotropic hypogonadism with weight gain, clearly aligning it with functional hypogonadism (40). This is similar to studies of US Army Rangers for their field training – under severe physical and psychological stress with the ambient diet not meeting their energy needs (41, 42). Rapid recovery of the HPG occurs, often within a week of refeeding and decreased stress. Adolescent boys and girls are at risk for this condition, whether athletes or not and whether post-pubertal or more relevantly, as a condition in which pubertal maturation is delayed in its entirety or its progress slowed.

## Systemic diseases

Outside the context of very poor general health status, chronic illness may affect the hypothalamic–pituitary level or the gonads or both. Many different mechanisms and modalities could be involved, according to the pathogenesis of the underlying systemic disease. Thus, unhealthy teenagers tend to have lower serum testosterone levels than age-matched healthy controls in the presence of normal or reduced LH levels, consistent with FHH (43).

### Chronic kidney disease (CKD)

Males and females with CKD both have increased LH and FSH levels. Young males have less testosterone and adrenal androgens and female teenagers have less estrogen and loss of LH pulsatile pattern (44). A cohort of 286 girls with CKD showed that 10% had delayed menarche, and 65 girls with delayed menarche had short stature compared with only 35% of girls without delayed menarche (45). Similar to treating girls with the Turner syndrome with estrogen, beginning early, approximately ages 11 or 12, had no effect on the near adult height of these adolescents, but a positive one on their quality of life (45).

### Iron overload

Diseases, for example, thalassemia major and hemochromatosis, causing iron overload can affect several endocrine organs. The pathogenetic mechanism involves the deposition of iron in excess within the endocrine tissues. Hypogonadism with iron overload due to transfusions is one of the most common endocrine dysfunctions and is mainly due to iron deposition within the gonads and/or the pituitary (46). Delayed or prolonged pubertal maturation is common in those with thalassemia major who have excessive body stores of iron. DeSantis and colleagues noted early puberty in boys with thalassemia major, but delayed testicular volume increase or progression of breast development along with a markedly slowed pubertal growth spurt. In adolescents and adults, the prevalence of hypogonadism has been noted as 43% in males and 38% in females (47, 48).

### Sickle cell disease

Worldwide, sickle cell disease (SCD), in those who have at least one copy of the hemoglobin S gene along with that for another abnormal hemoglobin, is a relatively common cause of delayed growth and pubertal maturation. Prior to 1970, few patients with SCD survived into adulthood and those that did had severe growth retardation and pubertal delay (49). Over the past 50 years, the combination of pneumococcal vaccine, universal screening of the newborn, early penicillin prophylaxis and hydroxyurea treatment has greatly extended the life span of these children as well as noted that their growth and pubertal maturation has increased but not quite yet to that of the general population (49, 50).

### Duchenne muscular dystrophy

Duchenne muscular dystrophy (DMD, OMIM # 310200) is an X-linked recessive disorder with a prevalence of 4.78 per 100,000 males (51), caused by mutations of the DMD gene, which is expressed in muscle sarcolemma and encodes the muscle protein, dystrophin. A small percent of the boys will have a contiguous gene deletion including the dystrophin and SHOX genes and thus would be expected to be shorter based on the latter gene on the X chromosome. Glucocorticoids are currently an almost universal component of the treatment of boys with DMD as they prolong mobility, preserve respiratory and cardiac function, reduce the need for surgery and increase lifespan; however, their use is complicated by adverse effects on growth, puberty and bone health with secondary osteoporosis and suppression of the HPG axis and adrenal androgen production (52). Historically, most boys naïve to glucocorticoid progressed through puberty normally; however, boys with DMD on long-term glucocorticoid therapy had significant pubertal delay (53, 54). Testosterone treatment has been associated with an increase in bone mineral density (55) and improved psychosocial outlook and self-image (56).

### Juvenile idiopathic arthritis

Juvenile idiopathic arthritis (JIA) is one of the most prevalent chronic diseases in children. DP is common in children with JIA, but as in many other conditions, it is the slowing of the pace of growth, pubertal maturation and prolonged duration or diminished growth spurt that are prominent (57). Endogenous and exogenous factors, for example, genes, nutrition, severity of the inflammatory state and glucocorticoid therapy, as well as psychosocial ones, can affect the timing and tempo of pubertal maturation. The pro-inflammatory cytokines, especially tumor necrosis factor α, interleukin 1β (IL), and IL-6 are prominent and may act directly at the growth plate (58). Immunomodulatory drugs show promise for the amelioration of symptoms and partial reversal of the growth retardation, often in concert with a lowering of the dose of glucocorticoids. Increases in bone mineral density accompany the decrease in the cytokines and glucocorticoid dose (58, 59).

In a study of 83 adolescent females with JIA, D’Angelo and colleagues noted a later timing of menarche when comparing the age at menarche to that of their mother or the normal population of Italian girls (60). The same investigators also noted that those with a systemic onset of signs and symptoms had less pubertal delay than those with oligoarticular or polyarticular disease (60). Other investigators described a 15% prevalence of DP in children with JIA compared to controls (1.4%). Their data also indicated an association of DP in boys with glucocorticoid dose and age at the start of the treatment (61).

### Crohn's disease (CD)

CS is a chronic inflammatory condition of the gastrointestinal tract, which may involve any part of the gastrointestinal tract, from the oropharynx to the perianal area. Pubertal delay and growth failure can be the initial presenting problems in the absence of obvious gastrointestinal symptoms (62). Data from the mid-1990s showed that the onset of breast development was delayed by 1.5 years in 75% of girls with CD (63, 64), whereas testicular development was delayed by almost a year in boys with CD (63). Despite modern therapy, a contemporary, retrospective study identified persistent pubertal delay in adolescents with CD, demonstrated by delay in age of peak height velocity (65). A more recent prospective study including clinical evaluation of puberty by a pediatric endocrinologist demonstrated that pubertal delay is still observed in adolescent girls. In boys, PD was not as common, but a diminished pubertal growth spurt was observed (66).

## Drugs

Many drugs such as opioids or glucocorticoids, alcohol abuse or illicit drug intake can cause acquired hypogonadotropic hypogonadism. Table 2 displays many of the common drugs that could potentially trigger hypogonadism.

**Table 2:** Medications potentially lowering serum testosterone levels.

Glucocorticoids
Antihypertensive drugs: sprinolactone and atenolol
Opiates
Endocrine agents: anabolic agents, GnRH analogs and 5 alpha-reductase inhibitors
Adrenal enzymatic suppressors: ketoconazole and metyrapone
Drugs increasing prolactin: antipsychotics
Chemotherapy and/or radiotherapy for malignancies

### Opioids

Substance abuse disorders affect approximately 2 million Americans, with more than 47,000 dying annually from a combination of heroin, fentanyl and other opioids (67). Secondary hypogonadotropic hypogonadism is likely caused by the various opioid receptors (mu, delta and kappa) on hypothalamic cells that eventually affect pulsatile GnRH release. In men, circulating testosterone concentrations may diminish within hours of opioid ingestion (68). Despite opioid-induced androgen deficiency being a well-established clinical condition affecting male patients, much is still unknown about the mechanisms and the clinical outcomes of chronic use of opioids and their analogs on male teenagers and the female HPG axis. In a case–control study (69), a significant increase in amenorrhea was observed in premenopausal women receiving either oral or transdermal opioids associated with a decrease in serum gonadotropin levels.

Analgesics, for example, acetaminophen, acetylsalicylic acid and ibuprofen are another class of agents that may directly affect testicular function. For ibuprofen, one controlled trial showed a significant decrease in the free testosterone/LH ratio after 2 weeks of drug ingestion. Those receiving ibuprofen also had a decline in their AMH levels, indicating that both testicular compartments, Leydig and Sertoli cells, are negatively impacted (70).

### Attention-deficit/hyperactivity disorder (ADHD) and stimulant medications

Attention-deficit/hyperactivity disorder is a prevalent condition among pre-pubertal and pubertal children. Stimulant medications with methylphenidate in many forms are commonly used therapeutically with some long-term concerns about height velocity and near adult height (71). A recent systematic review and meta-analysis of those children receiving methylphenidate showed a rather small decrement in height (SMD 0.27) and weight (SMD 0.33) and likely little clinical impact. The major effect was in the first 2 years of therapy but seemingly without a dose effect. The effect size for pubertal delay was very small derived from limited data (71).

### Anabolic steroids

Testosterone and many synthetic analogs form a large class denoted as anabolic–androgenic steroids (AAS). Contrary to popular belief, most AAS users are not competitive athletes but simply adolescents and young men who use these drugs to enhance personal appearance, sometimes because of underlying body image disorders ‘muscle dysmophia’ (72). The HPG axis is downregulated due to the negative feedback of testosterone or perhaps after its conversion to estradiol on GnRH and the gonadotropins, often accompanied with testicular shrinkage. In a single meta-analysis of 33 eligible studies including almost 3900 subjects with a few females, those who received AAS showed decreases in circulating levels of LH and testosterone. Despite the gonadotropin levels returning to within the normal range after 13–24 weeks, the serum testosterone levels continued to be suppressed after more than 4 months (73).

### Substance abuse

**Figure 1:**
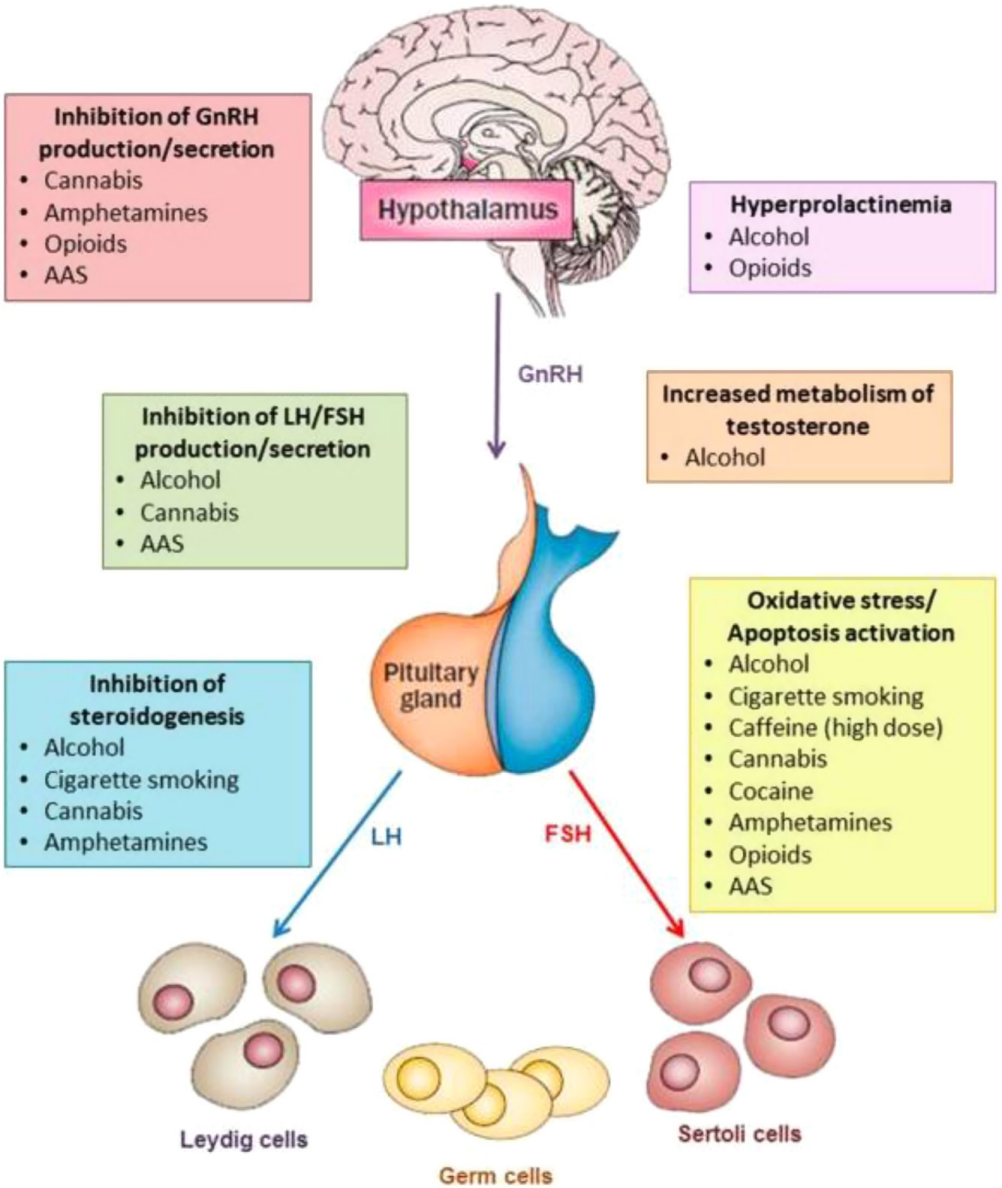
The main mechanisms by which substance/drug abuse may decrease testosterone levels and sperm production are inhibition of GnRH production/secretion, increase in prolactin levels, inhibition of gonadotropin production/secretion, inhibition of steroidogenesis, increase in testosterone metabolism, oxidative stress and induction of apoptosis. AAS, anabolic–androgenic steroids; FSH, follicle-stimulating hormone; GnRH, gonadotropin-releasing hormone; LH, luteinizing hormone (74).

Among unhealthy behaviors, alcohol abuse, cigarette smoking, excessive caffeine intake and illicit drug intake have been studied as a possible cause of reduced sperm production and/or reduced testosterone levels in hypogonadal men (74, 75) (Fig. 1).

Alcohol abuse may begin at a very young age. Recent US national data indicate that 9.7% of eighth-grade students and 21.5% of those in tenth grade have consumed alcohol within the past month (76). Besides the risk of developing alcohol use disorder, early alcohol consumption can result in delayed pubertal maturation (77). Alcohol use by adolescent boys causes suppressed serum levels of growth hormone (GH), LH and testosterone. In adolescent girls, alcohol use caused suppressed serum GH and estradiol levels (78). More recently, studies in girls have shown that prepubertal alcohol use was associated with delayed breast development (79) and delayed menarche (80). For the girls, delayed pubertal maturation was almost four times as common in those who consumed alcohol compared to those who did not (79).

A single case report found DP and low testosterone level in an adolescent male who heavily smoked cannabis. Following the discontinuation of cannabis, the testosterone levels increased and pubertal maturation with a linear growth spurt ensued (81). Spermatic function is diminished in adult males who use chronically abuse cannabis when they are compared to a matched control group, which does not (82).

## Initial evaluation

**Figure 2:**
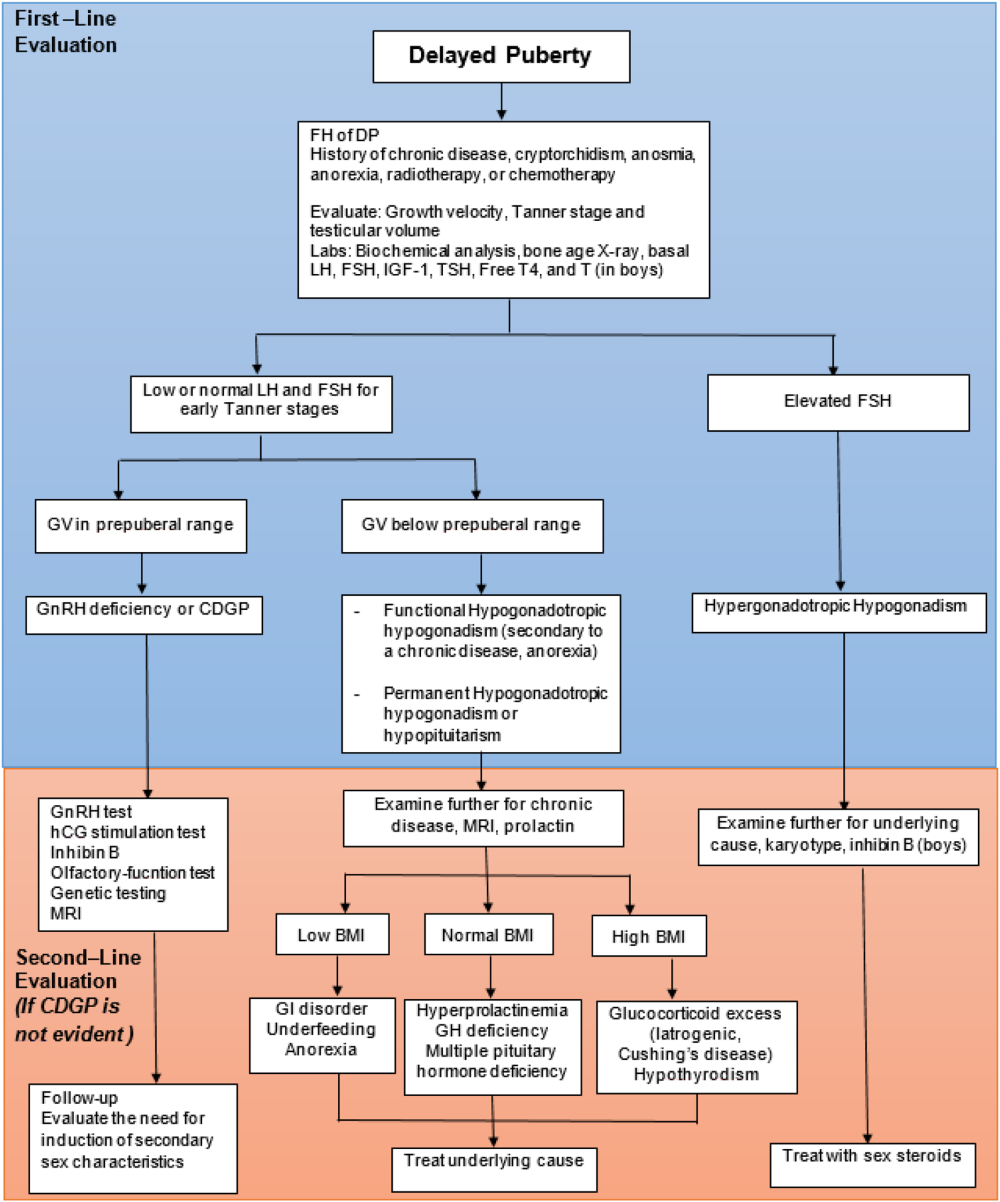
Algorithm for the evaluation of a patient with delayed puberty. BMI, body mass index; CDGP, constitutional delay of growth and puberty; FSH, follicle-stimulating hormone; GH, growth hormone; GI, gastrointestinal; GnRH, gonadotropin-releasing hormone; hCG, human chorionic gonadotropin; IGF-1, insulin-like growth factor 1; LH, luteinizing hormone; MRI, magnetic resonance imaging (3). From *New England Journal of Medicine*, Palmert MR & Dunkel L, ‘Delayed puberty’, Volume 366, Pages 443–453. Copyright © (2012) Massachusetts Medical Society. Reprinted with permission from Massachusetts Medical Society.

The absence of pathological medical history, signs and symptoms and a positive family history of pubertal delay (most commonly known as ‘late bloomers’) in one or both of the parents suggests a diagnosis of self-limited DP; however, before making the diagnosis, significant pathological conditions must be excluded. A thorough personal history should note evidence of chronic disease, anorexia, the intensity of exercise training and the timing of puberty of both parents. Chronic systemic disease or even a history of one is compatible with delayed pubertal maturation, although these delays are more often temporary than permanent (83). The presence or absence of ‘red flag’ features in the history and physical examination remains the strongest discriminator between isolated DP and hypogonadotropic hypogonadism. These include microorchidism, cryptorchidism or micropenis, indicating a lack of prior ‘mini-puberty’ or the presence of other features of GnRH deficiency, which include anosmia or hyposmia due to hypoplasia of the olfactory bulbs (in Kallmann syndrome) and occasionally cleft lip and palate, unilateral renal agenesis, short metacarpals, sensorineural hearing loss, synkinesia and color blindness (83). Initial evaluation (Fig. 2) will have likely been done in the primary care physician, family medicine or teen health provider, and then second-line evaluation where further assessment is required should be done in pediatric endocrinologist’s office. A karyotype should be performed, if a chromosomal abnormality, such as Turner syndrome is suspected and/or if the gonadotropin levels are elevated. If gonadotropin concentrations are elevated and primary ovarian failure is diagnosed, other testing would be required including autoimmune antibodies and Fragile X testing (84).

To minimize the biological variability in circulating testosterone concentration to improve diagnostic precision, we place a high value in recommending that testosterone should be measured in the fasting state between 07:00 and 11:00 on at least two different days. Measurement of testosterone during acute illness should be avoided. It is far easier to choose the testosterone assay limits for an adult than for adolescents during the pubertal maturation phase. For an adult male, a diagnosis of hypogonadism is highly unlikely with testosterone values >12 nmol/L (>350 ng/dL), but more likely in men with testosterone concentrations consistently <8 nmol/L (<231 ng/dL). The lower the testosterone concentration below the lower limit of the reference range, the higher is the likelihood that *symptoms* will be explained by testosterone deficiency. In those patients with borderline testosterone concentrations between 8 and 12 nmol/L (231–350 ng/dL), a clear diagnosis cannot be confidently established, but free testosterone measurements may often be helpful in this situation (85).

An additional difficulty may arise with the slowing height velocity just before the takeoff of the pubertal growth spurt. That is also a cardinal manifestation of GH deficiency and one must consider that within the differential diagnosis. When in doubt, it may be prudent to perform a GH stimulation test with two secretagogues but after appropriate sex hormone priming in these adolescent patients (86).

During pubertal maturation, there is often a wide variation of testosterone levels within most of the Tanner stages of maturation because often one does not know how long the adolescent was in that stage, that is, are the levels of testosterone the same for an early genital stage 3 and a late genital stage 3 adolescent – very likely not. At the same time, the day–night differences for mid-pubertal boys may be greatly magnified compared to the adult (87). For girls, the situation is made more difficult because of ovarian cycles, which occur, in young girls, even well before menarche (88). Consequently, these results should be interpreted carefully.

## Treatment

The management of delayed growth and especially delayed pubertal maturation is not so straightforward in those with FHH. The major target may be inflammatory cytokines, for example, in those with JIA or CD or trying to decrease the glucocorticoid dose as much as possible in those with DMD or other conditions for which glucocorticoids are prescribed. There are a number of psychosocial aspects that the patient and family along with the physician must consider; for some adolescent’s low-dose gonadal steroid hormone treatment whether only for the short term or more permanently is an important addition to the general disease-related therapy. In general, this should not differ from that for those with isolated pubertal delay, whether transient (CDGP) (16, 89) or permanent (hyper- or hypogonadotropic hypogonadism). There are some data to suggest that an early morning testosterone level may predict the onset of pubertal development in boys (90) and that even a short-term trial of low-dose testosterone therapy might serve as a diagnostic tool to predict the onset of puberty in boys (91). However, neither has been extensively studied in those with FHH. Escalating doses of testosterone, at present, mainly delivered intramuscularly or subcutaneously (other forms of delivery are under investigation), are the mainstay for boys and have been reviewed recently (7, 92). There are fewer data for girls with most coming from the treatment of girls with the Turner syndrome (93, 94, 95).

## Summary and implications

DP is a frequently encountered condition; however, the most common etiology is self-limited, CDGP. The differential diagnosis includes hypogonadotropic hypogonadism, primary hypogonadism and functional hypogonadism, which must be considered in adolescents with DP. Hypogonadism and its medical and psychological consequences can be underestimated or overlooked in patients with chronic illness, resulting in clinical under management and poorer quality of life for the adolescent. This requires careful consideration and expertise by health-care providers who manage adolescents especially during the expected pubertal maturational period and as they approach the transitional period to emerging adulthood.

## Declaration of interest

ADR consults for Antares Pharma, Ascendis Pharma, BioMarin Pharmaceutical, Lumos Pharma, Pfizer, Tolmar Pharmaceuticals, and the United States Anti-Doping Agency (USADA). He receives royalties from UpToDate. RE-Z has nothing to declare.

## Funding

There was no external funding for this project.

## Author contribution statement

RE-Z and ADR contributed to the original outline and wrote multiple versions before agreeing to the final version.

## Data availability statement

There were no data generated for this report.
